# Alzheimer’s Disease Biomarker Detection Using Field Effect Transistor-Based Biosensor

**DOI:** 10.3390/bios13110987

**Published:** 2023-11-17

**Authors:** Phan Gia Le, Seong Hye Choi, Sungbo Cho

**Affiliations:** 1 Department of Electronic Engineering, Gachon University, Seongnam-si 13120, Republic of Korea; legiaphan2020@gachon.ac.kr; 2 Department of Neurology, College of Medicine, Inha University, Incheon 22332, Republic of Korea; 3 Gachon Advanced Institute for Health Sciences and Technology (GAIHST), Gachon University, Incheon 21999, Republic of Korea

**Keywords:** field-effect transistor (FET), electrochemical sensor, Alzheimer’s disease, biomarkers, amyloid beta, tau

## Abstract

Alzheimer’s disease (AD) is closely related to neurodegeneration, leading to dementia and cognitive impairment, especially in people aged > 65 years old. The detection of biomarkers plays a pivotal role in the diagnosis and treatment of AD, particularly at the onset stage. Field-effect transistor (FET)-based sensors are emerging devices that have drawn considerable attention due to their crucial ability to recognize various biomarkers at ultra-low concentrations. Thus, FET is broadly manipulated for AD biomarker detection. In this review, an overview of typical FET features and their operational mechanisms is described in detail. In addition, a summary of AD biomarker detection and the applicability of FET biosensors in this research field are outlined and discussed. Furthermore, the trends and future prospects of FET devices in AD diagnostic applications are also discussed.

## 1. Introduction

Alzheimer’s disease (AD) is associated with neurodegeneration and causes many symptoms in the aging brain, including cognitive impairment [1]. Many patients over 65 years old with AD directly impact life and society, especially in developed countries [2]. Thus, remediation for AD prevention and treatment is urgently needed.

Several aspects of AD have been studied and developed, including its mechanisms, biomarker detection, clinical research, and drug delivery for diagnosis and treatment [1,3]. However, many drawbacks have led to less effective application in the progression of AD, especially at the onset stage.

Nowadays, amyloid-beta (Aβ), phosphorylated tau (p-tau), and total-tau (t-tau) biomarkers are broadly approved as fingerprints of AD [4,5,6]. In contrast, few studies are working on acetylcholine [7,8] and α-synuclein [9]. Previous longitudinal and cross-sectional research has indicated that prior to showing AD symptoms, the levels of Aβ_1-42_, p-tau, and t-tau start to change after roughly 10–15 years [10,11]. A high concentration of p-tau in the blood indicates neurodegeneration in the brain, which ultimately leads to AD [12]. Aβ_1-42_ is more toxic than that of Aβ_1-40_ due to its faster formation pace of aggregation. An estimation of the Aβ_1-42_ level can detect the formation of AD, while the Aβ_1-42_ to Aβ_1-40_ ratio provides further information on the current stage of AD progression [13]. An alternation of α-synuclein level is associated with brain Aβ-plaque deposition and p-tau_181_ in AD [14,15]. Acetylcholine (Ach), a neurotransmitter, intimately connects to the neural signal transmission, and a deficiency of Ach concentration production induces AD progression [8]. To detect these AD biomarkers, a variety of methods has been employed, from conventional methods, such as positron emission tomography (PET) [16], magnetic resonance imaging (MRI) [17], and near-infrared fluorescence (NIRF) [18], to modern methods, such as electrochemistry [19,20], fluorescence [21], and colorimetry [22,23]. Each of these provides a particular insight into AD and has advantages and disadvantages. Conventional methods provide intuitive observation at low spatial resolution [16,17,18], and optical methods generally have trouble-free operation and visualization at a low limit of detection (LOD) [22,23,24]. A combination of optical methods effectively improves the LOD [25]. Electrochemical sensors have attracted considerable attention due to their real-time detection, rapid response, and ultrasensitivity [26,27] compared to their counterparts, and have been applied in various research areas, such as environmental monitoring, food safety, and medical fields. Currently, biosensors are used for biomarker detection in several diseases, such as diabetes [28,29], cancer [30,31], Parkinson’s disease [32,33], and AD [34,35]. Biomarker detection of AD using an electrochemical sensor has achieved impressive results at an exceptionally low LOD of pico- or femtomole [26,27], promoting early recognition of the AD signal for diagnosis and treatment.

Recently, a diversely miniaturized electrochemical device was designed, fabricated, and practically applied, which showed an effective approach to obtaining the ultrasensitive detection limit of AD biomarkers, such as two-electrode [27], three-electrode [36], interdigitated electrode [37,38], and field-effect transistor (FET) systems. Among these, an electrochemical system with two or three working electrodes is often used. In contrast, FET biosensors are emerging devices that have drawn much attention due to their wide range of applications and ultra-sensitivity down to pico- or atto-moles per liter [39]. With the proper design of FET-based biosensors, real-time detection, precise detection of target molecules, an ultrasensitive detection limit, insignificant dimensions due to compact integration, low expenditure, and mass production ability are achieved [8,40], which provides a suitable application of FET-based biosensors for AD biomarker recognition in both qualitative and quantitative research. There are several review papers focusing on AD biomarker detection. Among these articles, AD biomarkers were recognized by numerous emerging techniques [41,42], or just concentrating on one kind of sensor [43]. To the best of our knowledge, there has been no publication that overviewed all AD biomarkers detected by utilizing FET biosensors. Thus, it will be interesting to contribute a review paper relying on such content to broad audiences.

In this review, the relevant features of FET-based biosensors and their application in AD biomarker detection are discussed in detail. In addition, multiple strategies for sensor design and fabrication were overviewed and analyzed in both aspects of physical foundation and biomarker detection. Finally, the current challenges and future vision for commercialization of the FET biosensor application in AD diagnosis are also discussed.

## 2. Overview of the FET Biosensor

### 2.1. The FET Biosensor Architecture

**Figure 1:**
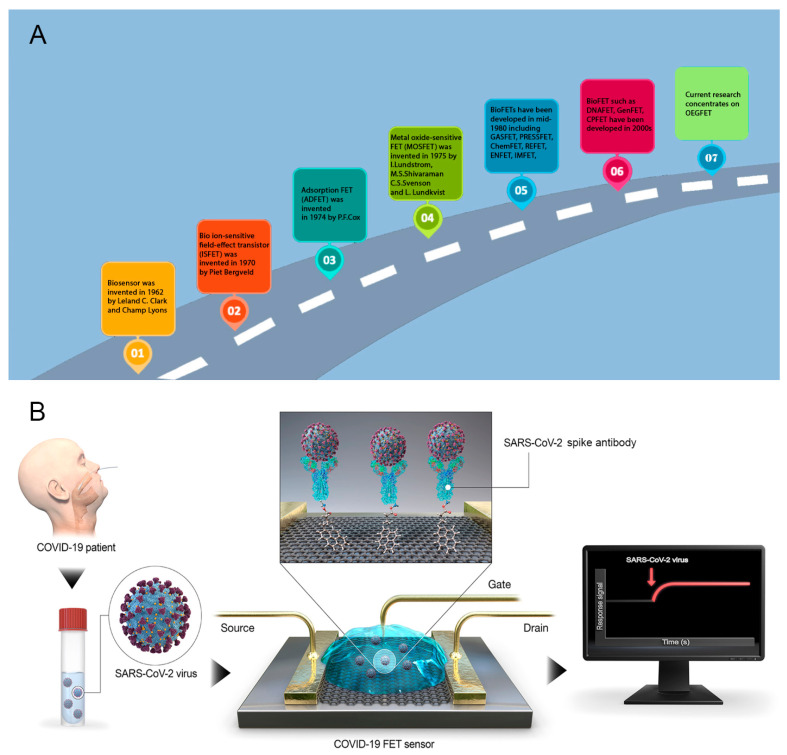
(**A**) A roadmap describing the progress in the development of field-effect transistor-based biosensors [54,55,56,57,58]; (**B**) Architecture of the representative FET biosensor [59].

Architecturally, a typical FET biosensor is composed of three electrodes: drain (D), source (S), and gate [44,45], and a roadmap for FET biosensor development is illustrated in Figure 1. Under an applied voltage, current flows from the source to the drain electrodes. Gate electrodes are typically classified as back [46], top [47], floating [48,49], and solution gates [50,51]. Gate electrodes play a vital role in FET biosensors; by reducing the accumulation of electron density in the fluidic channel, the gate electrode can adjust the conductance of the channel and stabilize the electrical signal [52,53]. Channel conductance depends on the correlation between the target molecule charge and the type of semiconductor; for example, an increase in conductance occurs with an n-type semiconductor and positively charged target molecules. Similarly, the opposite trend was observed for p-type semiconductors [52].

### 2.2. Nanomaterial Preparation for FET Sensor Fabrication

**Figure 2:**
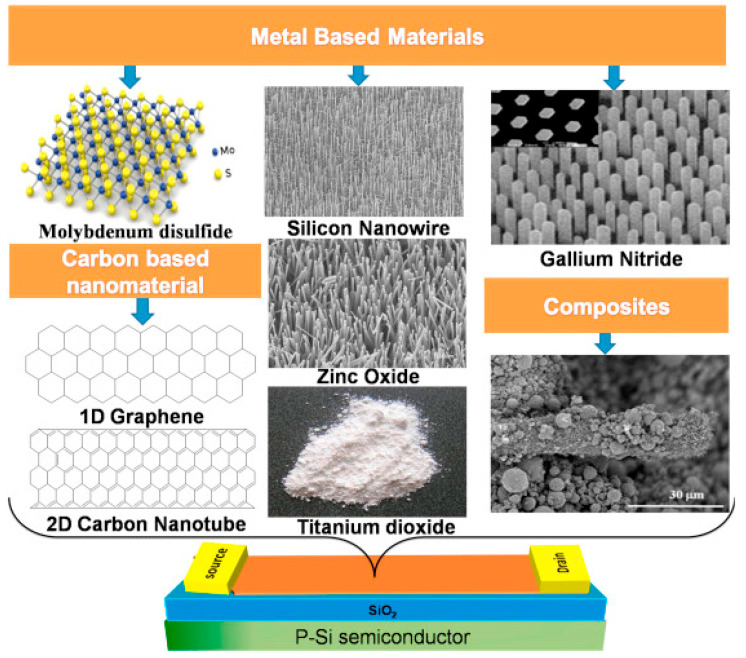
Various carbon- and metal-based nanomaterials coated on the channel layer surface of the FET biosensor [86].

To manufacture a FET biosensor, a wide range of nanomaterials has been applied, namely, oxides [44,45], carbon [47,60], conductive polymers [61,62], and composites [63,64] with diverse morphologies, such as nanoparticles [65], nanorods [66], 2D nanosheets [47], nanotubes [67,68], and nanowires [69] due to their high surface area to volume ratio, which is beneficial for immobilizing a large number of biological receptors, as illustrated in Figure 2. The existence of immobilized receptors in the device enables FET biosensors to recognize biomarkers, namely, prostate-specific antigens [70,71], antibiotics [72,73], bacteria [74,75], and viruses [76,77,78], with high sensitivity and selectivity. FET biosensors are classified into n- and p-type devices based on the type of semiconductor used for channel fabrication [79,80]. Many advanced techniques have been widely applied to transfer materials to the sensing area of devices, such as chemical vapor deposition (CVD) [81,82], atomic layer deposition (ALD) [83,84], spin coating [85], photolithography [9,39], and Langmuir–Blodgett (LB).

**Figure 3:**
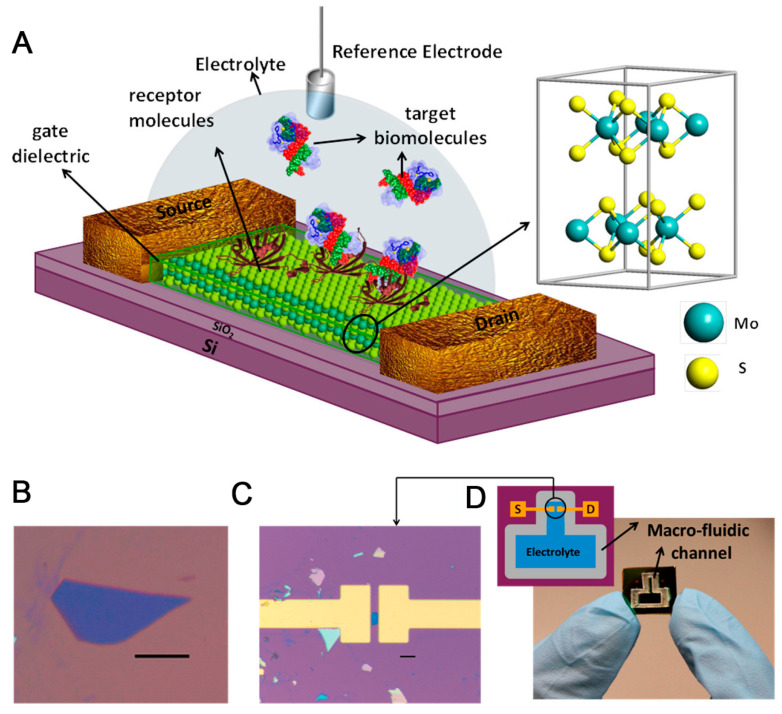
(**A**) Illustration of the MoS_2_-based FET biosensor for pH quantification; (**B**) Optical image of MoS_2_ flake on SiO_2_; (**C**) Optical image of MoS_2_ FET biosensor with extended electrodes made of Ti/Au; (**D**) Image and schematic diagram of chip with the biosensor device and microfluidic channel [91].

A representative example of metal oxide-based FET biosensors is MoS_2_ material, a semiconductor with typical features, including a bandgap of ~1.9 eV for monolayer and 1.29 eV for multiple layers, transparency, and flexibility [44,87]. In MoS_2_-based FET, the MoS_2_ thin film is prepared using the CVD method [87,88]; however, this method usually produces many sulfur defects in the structure, resulting in an n-type MoS_2_ semiconductor as the final product. To fabricate p-type MoS_2_ semiconductors, N-doped MoS_2_ was fabricated using N plasma flowing through a thin film [87]. MoS_2_ in the device, with its intrinsic characteristics, enables the detection of H_2_O_2_ [89] and glucose [90]. Furthermore, the combination of a MoS_2_-FET device functionalized with receptors or doping can detect diverse target molecules, as demonstrated in Figure 3.

**Figure 4:**
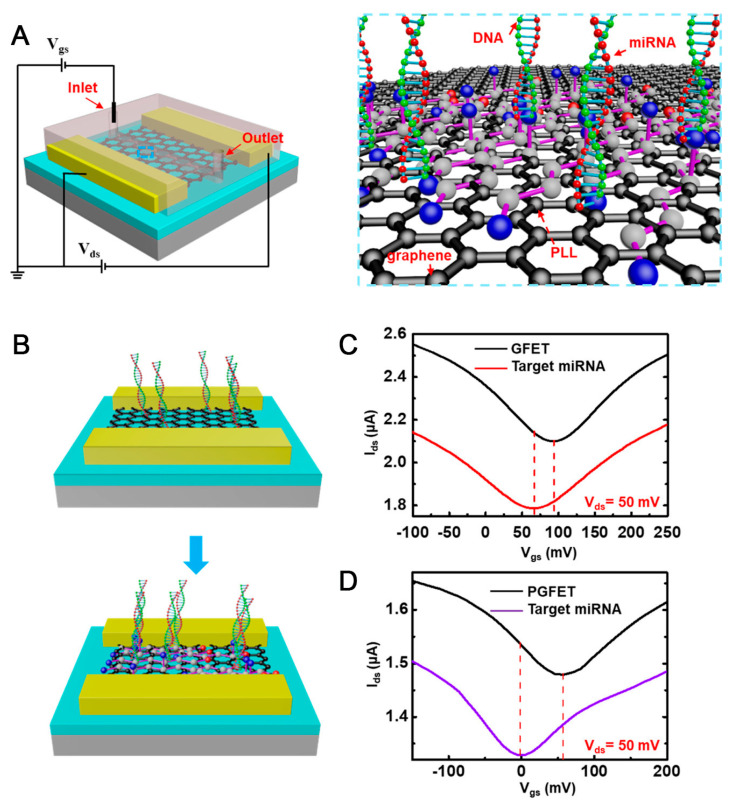
(**A**) Illustration of the graphene-based FET biosensor for breast cancer miRNAs and SARS-CoV-2 RNA detection; (**B**) Schematic principles of graphene field-effect transistor (GFET) and poly-l-lysine (PLL)-functionalized graphene field-effect transistor (PGFET) for miRNA detection; (**C**,**D**) miRNA detection results for GFET and PGFET, respectively [100].

The 2D sheet graphene oxide material-based FET biosensor is a typical example of a carbon material that is well-known for its nanostructure, few-nanometer thickness, high specific surface area, high electron transfer, and flexibility [92]. In addition, graphene oxide has been used in a broad range of applications, including batteries, catalysts, and sensors [93]. In FET biosensors, graphene has been utilized for channel layer fabrication [45,94]. Furthermore, a large number of hydroxyl (-OH) and carboxyl (-COOH) functional groups on the surface make graphene oxide act as an anchoring point for functionalizing other chemical molecules or linkers, facilitating biological immobilization [94,95]. In addition, modified graphene exhibits a strong change in its intrinsic characteristics and forms a new type of structure with extraordinary features [96,97] that are beneficial for the biological application of FET biosensors, as depicted in Figure 4. As described above, popular materials for FET biosensor fabrication include MoS_2_ and graphene oxide. Other materials, including SiO_2_ and Au, are widely utilized. These materials are a foundation for biological receptor conjugation through the construction of a transitional self-assemble monolayer (SAM) [2]. This approach can be applied by treating pristine materials to obtain hydroxylated or thiolated surfaces [98,99].

The materials applied to FET biosensors are diverse and abundant. Surface modification is an effective approach for achieving highly impressive performance and has been used in many applications [92,101]. The choice of specific materials for the desired FET biosensors depends on the strategy and availability of the apparatus and the purpose of the practical application. Advanced materials and specialized tailoring substantially contribute to design and performance improvement.

### 2.3. Overview of Sensing Mechanism of FET Biosensor

**Figure 5:**
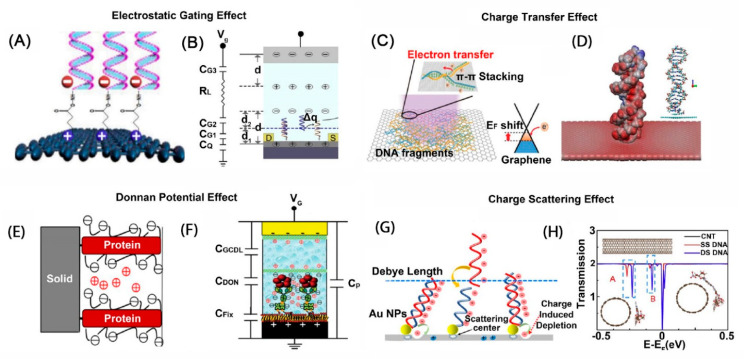
Illustration of different sensing mechanisms of graphene-based FET biosensor [107]: (**A**,**B**) Electrostatic gating effect; (**C**,**D**) Charge transfer effect; (**E**,**F**) Donnan potential effect; (**G**,**H**) Charge scattering effect.

The sensing mechanism of an FET biosensor is classified into four categories, such as electrostatic gating, charge transfer, Donnal potential, and charge scattering effects. In the electrostatic gating effect, the charges of the biomolecules trigger an opposite charge that presents on the sensing material, which changes the charge density and directly affects the electrical properties [102]. For example, a negatively charged phosphate group of immobilized DNA induced a p-doping graphene sensing material [102]. This effect could not explain the Dirac point shift, whereas the charge transfer effect could. The density functional theory expounds that multiple electrons will transfer from an aromatic group of immobilized DNA to graphene sensing material through π-π bonds [103]. As a result, by making a change to the sensing material charge density, the charge carriers will be redistributed with an increase in negative charge. A competitive mechanism between the electrostatic gating and charge transfer effects was also proposed; this correlation decides whether the charge density will increase or not [95]. The electrostatic gating effect is only explained for biomolecules having Debye length, whereas it fails to explain for others beyond the Debye length scope [104]. However, the Donnal potential effect can be utilized to explain this purpose. The formation of a semi-permeable membrane between the bulk solution and biomolecular layer prevents other charged biomolecules on the biomolecular layer from penetrating inside, and therefore a difference in the electrical field between the two faces of the semi-permeable membrane establishes a Donnal potential [105]. In the charge scattering effect, the presence of charges on the surface of the sensing material causes the scattering effect between them and the charges in the sensing material, leading to a reduction in conductivity, and inducing a negative response current [106]. These mechanisms can be illustrated, as in Figure 5, and more detailed ones are presented elsewhere.

### 2.4. Responsive Signal of FET Biosensor

Based on the sensing mechanism, three kinds of signals were broadly studied, including voltage, current, and capacitance responses. The charge transfer effect causes a redistribution of the charge carrier on the sensing materials, accompanying a shifting Dirac voltage as well as a shift in threshold voltage [102,108]. Besides the voltage response, the source–drain current response is also more widely employed in FET biosensors as a subsequence of the charge scattering effect, and therefore various magnitudes of the responsive current can be recorded [39,109]. Moreover, capacitance response is also utilized as a responsive signal through the Donnal potential effect [110,111].

## 3. Application of FET Biosensors in AD Biomarker Detection

### 3.1. AD Biomarker Detection Methodology

As aforementioned, Aβ and tau are two key biomarkers widely approved and extensively studied. The presence of electroactive groups, such as tyrosine, histidine, and methionine, in the Aβ structure enables the detection of this biomarker by recognizing oxidized peaks at ~0.6 V and 1.5 V, and waves at 1–1.5 V, respectively [109,112,113]. However, this approach is non-specific. Thus, a highly specific method has been employed to circumvent this bottleneck and manipulate the antigen–antibody immunoreaction [34,35]. Thus, the Aβ biomarker of AD has been detected with high specificity. In contrast, the non-specific surface area was blocked by bovine serum albumin (BSA). Many peptide chains of Aβ have existed, among these, Aβ_1-40_ and Aβ_1-42_, have drawn much attention. Previous articles have indicated Aβ_1-40_ and Aβ_1-42_ are not toxic in monomeric states, and vice versa in oligomeric states. An estimation of monomeric Aβ and oligomeric Aβ concentration provides information on AD at onset and further stages, respectively. Noticeably, AβO can be divided into toxic and non-toxic groups, and the Aβ toxic oligomer-selected sensor can be produced by utilizing the anti-AβO NU4, anti-amyloid A11, and greater than 50 kDa, prion protein (P_r_P^c^), poly(curcumin-Ni) [114,115,116]. Similarly, a tau biomarker was detected to provide information for the diagnosis and treatment of AD. The tau biomarker was detected using anti-tau antibodies to achieve high specificity [35,81]. Besides Aβ and tau biomarkers, acetylcholine and α-synuclein are recognized and quantified by conjugating antibodies [8,9]. AD biomarker detection was conducted in phosphate-buffered saline (PBS), human serum (HS), human serum albumin (HSA), human blood (HB), saliva, and human plasma (HP). To be an effective aid for AD studies, research results must be fast and precise; thus, the FET sensor must be modified to obtain high selectivity, sensitivity, and specificity.

### 3.2. Recent Research Progress in AD Biomarker Detection

#### 3.2.1. Architecture of a Fabricated FET Biosensor through Recent Representative Research

Architecturally, FET biosensors for AD biomarker detection are similar to those designed for other target molecules, including source, drain, and gate electrodes, as mentioned in the previous section. The necessary substrates utilized for FET biosensors were fabricated from diverse materials, such as SiO_2_, Si, SiO_2_/Si, and Kapton [8,9,34,35,39,40,81,109]. Channel layers were made from Au, Al_2_O_3_, graphene (G), graphene oxide, reduced graphene oxide (rGO), and carbon nanotubes (CNT) [8,35,49,81,117]. 

Various advanced materials and techniques have been used to fabricate FET biosensors. In this section, FET biosensor physical substrates are discussed as the first foundation for further work. Ciou et al. [81] fabricated an FET biosensor in which a 6 nm film of Al_2_O_3_ was deposited on a Si substrate by ALD. Then, drain, source, and planar gate electrodes were generated by the thermal evaporation of Cr/Au (5/50 nm) through a shadow mask. Next, bilayer graphene (BG) was attached to the electrodes by thermal annealing, followed by low-damage plasma treatment to form a GO/G-layered composite on the Al_2_O_3_/Si substrate. Ti and Au thin-film layers were prepared on a glass slide substrate by thermal evaporation through a shadow mask [34]. The Au layer was continuously immersed in the piranha solution, rinsed with deionized water, dried with N_2_, treated with UV/ozone and O_2_ plasma, and then finished. The SiO_2_ substrate was occasionally treated in an oxygen–plasma environment as a modified substrate [34]. Kwon et al. developed an FET biosensor [35] in a 15 nm titanium adhesive layer, and 85 nm thick platinum was deposited on a SiO_2_/Si substrate using a lithography process. A graphene film was then formed on the Cu layer by CVD and coated with poly-methyl methacrylate (PMMA). After Cu etching, the PMMA/G was transferred onto the modified substrate and dried under ambient conditions. The PMMA was removed by washing with acetone and rinsing with 2-propanol. 

The FET biosensor was operated without a microfluidic channel combination, and the target molecule in a droplet of the main solution drifted to a higher concentration because of evaporation [9]. To overcome this limitation, FET biosensors have been incorporated with microfluidic channels [8,9]. According to Ricci et al., a FET biosensor was constructed by depositing a Au/Cr thin film on a Kapton surface [9]. A coplanar gate electrode was prepared by organic deposition and covered with dextran. Next, the source and drain contacts were immersed in 2,3,4,5,6-pentafluorothiophenol (PFBT) for functionalization. Subsequently, the gate electrode state was recovered by dextran removal. Chae et al. reported an FET biosensor [8] in which 100 nm thick Au, as the source electrode, was deposited on a SiO_2_ substrate by e-beam evaporation, followed by -NH_2_ group functionalization after treatment with piranha solution and soaking in 3-(ethoxydimethylsilyl)propylamine (APTES). A thin layer of GO was bonded onto the APTES-treated SiO_2_ substrate by spin coating, then proceeded with hydroiodic acid (HI) to produce rGO; Al_2_O_3_ was deposited as a sacrificial layer, and the Au electrode was passivated. Similarly, Park et al. [39] fabricated a slightly modified FET biosensor. In this study, APMES was employed to generate the -NH_2_ group instead of APTES. The rest of the FET fabrication resembled earlier work by etching to produce rGO thin films and attaching Ti/Au drain and source electrodes through a lift-off process.

#### 3.2.2. AD Biomarker Detection via Representative Research

The FET biosensor has been widely applied to detect AD biomarkers such as amyloid beta [34,109], phosphorylated tau (p-tau) [40,81], acetylcholine (Ach) [118], acetylcholine esterase (AChE) [8], and α-synuclein [9]. The reported detection environments include PBS, cell culture media, HSA, artificial cerebrospinal fluid (CSF), plasma, and clinical samples. Based on previous studies, electrode surfaces are mainly functionalized for enzyme immobilization [8], aptamers [13], and antibody conjugation [81]. In addition, functionalization can be performed by direct or indirect connections to transducers [35]. Detection can be performed with single, dual, or quad target molecules [39,117], and the obtained signal can be single [9] or combined [34].

**Figure 6:**
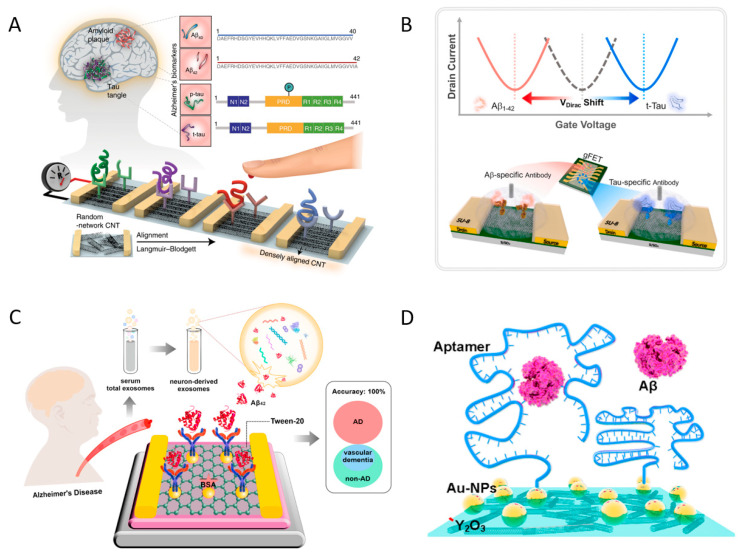
(**A**) A schematic illustration of the CNT-based FET sensor for simultaneous detection of various AD biomarkers [117]; (**B**) A schematic illustration of the graphene-based FET for multiplex detection of Aβ_1-42_ and t-tau [39]; (**C**) Schematic illustration of the G-EGT biosensor for NDE-Aβ_1-42_ detection [119]; (**D**) Illustration of aptamer probe immobilization onto FG using a typical Au linker, followed by Aβ peptide immobilization [49].

In the previous section, the physical foundations were separately discussed. In this section, the studies conducted on bioreceptor conjugation, which serves as a probe for biomarker detection, are discussed. For the Aβ_1-42_, Hideshima et al. functionalized APTES on the SiO_2_-treated substrate, which acted as anchorage for immobilizing congo red (CR) and anti-Aβ_1-42_ antibodies by utilizing glutaraldehyde as a cross-linker [34]. CR could strongly interact with Aβ_1-42_ fibrils, although it was not specific to the Aβ_1-42_ monomer or the Aβ_1-42_ oligomer. Thus, the detected signals in this work were generated by both CR and Aβ_1-42_ antibodies via total interaction. These results prove that CR immobilization was effective under the same reaction conditions. A new approach was described by Wustoni et al. [109], where CR was immobilized on the quaternized membrane utilizing glutaraldehyde as a cross-linker, which was the first time that CR and an isoporous membrane were utilized simultaneously to increase affinity toward Aβ_1-42_ aggregation. The fabricated biosensor was adopted to detect Aβ_1-42_ aggregation with a linear range of 2.21–221 nM and a sensitivity of 216 µA/dec. Kim et al. designed a special FET biosensor [117] that can detect Aβ_1-42_, Aβ_1-40_ t-tau, and p-tau_181_ simultaneously, as illustrated in Figure 6. In sensor manufacture, various antibodies were immobilized on sulfo-N-hydroxysulfosuccinimide (NHS)-functionalized CNT to detect those biomarkers with a linear range from 100 to 106 fM and LODs of 2.13, 2.20, 2.45, and 2.72 fM for Aβ_1-42_, Aβ_1-40_, t-tau, and p-tau_181_, respectively. By simultaneously detecting t-tau/Aβ_1-42_, p-tau/Aβ_1-42_, and Aβ_1-42_/Aβ_1-40_ in clinical plasma samples, the fabricated sensor could differentiate patients with AD from healthy controls. In addition to antibodies that act as probes, aptamers have been widely employed. For example, Kutovyi et al. designed a FET biosensor for Aβ_1-40_ detection based on a single-trap phenomenon approach [13]. In this study, modified single-stranded deoxyribonucleic acid (ssDNA) was functionalized with the assistance of 3- glycidyloxypropyltrimethoxysilane (GPTES) via incubation. The Aβ_1-40_ was recognized by capture time constant monitoring (single-trap approach) in novel Si two-layer (TL) NW FET structures with a linear detection range of 1–10 µg/mL and a detection limit of 20 fg/mL. The exposure sensitivity of the new technique was 300% higher than that of the conventional technique (drain current monitoring). In another study, Chen et al. fabricated an FET biosensor [49] with multiple-device integration, in which CNT were used as scaffolds and Au NPs as anchoring points for connecting DNA aptamers. Two kinds of biomarkers could be detected in the linear range of 1–10 pM at LODs of 45 and 55 aM, corresponding to Aβ_1-42_ and Aβ_1-40_, respectively. This sensor exhibited a high selectivity of up to 730% for Aβ_1-40_ and 800% for Aβ_1-42_, and recovery from 88% to 108% in the operating linear range. Salehirozveh et al. designed a reduced graphene oxide-based FET biosensor for Aβ_1-42_ detection with an RNA aptamer probe [108]. In this study, a Si/SiO_2_ substrate was activated by a piranha solution and then functionalized with APTES, and GO nanosheets were bound to the animated substrate, which was reduced by hydrazine to produce rGO. The modified surface was immobilized with an RNA aptamer for the detection of Aβ_1-42_. The fabricated sensor could detect Aβ_1-42_ in PBS with a linear range of 1 ng/mL to 1 pg/mL at neutral pH. Li et al. designed a reduced graphene oxide-based FET biosensor to detect SH-SY5Y-derived exosomal Aβ_1-42_ (NDE-Aβ_1-42_) utilizing the antifouling strategy with a dual blocking process [119]. In this sensor, Au NP was coated on the rGO surface. Then, thioglycolic acid was utilized to generate -COOH on Au NP, which was activated by the NHS/EDC for anti-Aβ_1-42_ antibody conjugation. Non-specific areas were blocked using dual agents: BSA and Tween-20. The sensor was first adopted for detecting standard Aβ_1-42_ in PBS with a linear range of 1.48–148 pg/mL at a LOD of 447 ag/mL and subsequently applied for NDE-Aβ_1-42_ detection in clinical sample detection.

García-Chamé et al. designed an FET biosensor [40] in which SAMs were made from COOH-EG8-thiol bonded to the Au surface; -COOH groups were activated by an EDC/NHS solution, and antibodies were conjugated through incubation. The generated sensor detected tau protein in artificial CSF with a sensitivity proportional to the mass of polyethylene glycol (PEG); specifically, the sensor made from 20 kDa PEG exhibited higher sensitivity than that made from 10 kDa PEG. The detection limits in the cell culture media and artificial CSF were less than 1 and 10 pM, respectively. Kwon et al. [35] directly or indirectly immobilized anti-tau antibodies onto pristine graphene or pyrenebutanoic acid succinimidyl ester (PSE or PBASE)-linked pristine graphene. The linear range of detection ranged from 10 fg/mL to 1 ng/mL, and the current change rate of the linker-free patterned graphene sensor was two to three-fold higher than that of the PSE linker-pristine graphene sensor. An FET biosensor could detect both β_1-42_ and total-tau (t-tau) proteins [39]. First, anti-Aβ_1-42_ and anti-t-tau antibodies were immobilized on the biosensors with the aid of PBASE through π-π stacking and functional group interaction. The FET biosensor was combined with a microfluidic channel chamber. This FET biosensor could detect Aβ_1-42_ and t-tau biomarkers with a linear range of 10^−1^–10^5^ pg/mL and a detection limit at the femtomolar level in PBS, human plasma, and spiked CSF. Ciou et al. [81] reported that antibodies were functionalized on the surface of a GO/G bilayer to detect the p-tau_217_ biomarker. As a result, a linear range of 10–100 pg/mL and sensitivity of 18.6 mV/dec in PBS were obtained. These values were slightly lower in human serum than those in PBS.

The reported FET biosensors were applied for Aβ and tau key biomarker detection. However, they were utilized for α-synuclein and acetylcholine detection. For example, Ricci et al. fabricated an FET biosensor for α-synuclein detection [9] with two approaches of anti-α-synuclein antibody conjugation and NH_2_-terminated SAM activated by glutaraldehyde and His-tagged recombinant protein G. As a result, the linear range of detection was from 0.25 pM to 25 nM, and detection limits were comparable at the sub-pico level for both approaches. Chae et al. reported an FET biosensor [8] in which rGO was functionalized with acetylcholinesterase using PBASE for Ach detection. Based on the fabricated biosensors, Ach was quantified with a linear range of 1–10 mM and a slope of 13.9 mV/dec on a logarithmic scale, which cover the scope of biomarker detection in spiked samples and clinical samples. Experiments on clinical samples are a further step toward sensor performance evaluation and a pre-step for commercialization. A summary of the representative FET biosensors is presented in Table 1. 

Although scientists have used diverse approaches, the fabricated FET biosensors have achieved an ultra-low detection limit in the picomolar range, which is appropriate for the detection of AD biomarkers. The ACh detection range, in particular, from micro- to millimole levels, needs improvement to align with a suitable detection range in practice.

#### 3.2.3. Signal Response of FET Biosensor via Representative Research

In this section, we will discuss the signal response of FET biosensors for AD biomarker detection from representative articles. Generally, two popular approaches for analyzing the behavior of the FET biosensors are the Dirac voltage (V_G_) point and source–drain current (I_SD_) responses [8,9,35,39,81,109], in which the I_SD_ vs. V_G_ response usually has a typical V-shape, while the I_SD_ vs. V_SD_ response is linear line shape. Chae et al. detected ACh by observing the I_SD_ alternation as a function of V_G_; the current response has a typical V-shaped curve with V_Dirac_ shifted to a positive value based on various V_G_, whereas the I_SD_ vs. V_SD_ response showed a linearly proportional or a good ohmic relationship [8]. Ricci et al. detected α-synuclein by relying on the relationship between I_SD_ and V_G_, in which V_SD_ = 0.1 V, and V_DS_ ranges from 100 to 400 mV. A fluctuated signal near the V_DS_ point was observed; however, this did not affect the V_Dirac_ shift trend [9]. Park et al. detected Aβ_1-42_ and t-tau by applying the I_SD_ vs. V_G_ response; a shift toward a positive voltage for p-doping and a negative voltage for n-doping materials in Aβ_1-42_ and t-tau cases was observed, respectively. The sensor acted identically for different environments of CSF and human plasma [39]. Besides their study on gate V_G_ and I_DS_, Kytovyi detected Aβ_1-40_ by utilizing the capacitance response [13]. In this work, capacitance was a function of liquid gate voltage, and the change in capacitance accompanied the change in recorded signals, making the AD biomarker detectable. Generally, approaches on the current and voltage are popular, while on capacitance is rare.

Besides the obtained average values in each experiment, the error bar is also to be considered as a crucial factor in biosensor study. Firstly, the error bars describe precise and reliable data after repeating measurements through average value and standard deviation. Secondly, the error bar proves the reproducibility of each specific experiment. In the studied articles, the error bar response is different. Some works displayed an increasing error bar from low to high analyte concentration [8,34,39], while others showed the opposite trend [40]. Some reports exhibited a small error bar [81], which proves the reliably measured data. In contrast, others presented a high value of the error bar even though it overlapped with the next average point [9,109], which reflected less precise data and was not reliable. Thus, experimentalists need to control reaction-related factors and necessary conditions to obtain good and trusted data.

## 4. Conclusions and Future Vision

FET biosensors are ready to proceed down the path of commercialization if several factors are achieved, such as sensitivity, selectivity, reproducibility, scalability, low-cost production, and continuous signal monitoring. Previous publications have revealed that FET biosensors are sensitive enough (at femto- or picomolar concentration) to detect AD biomarkers. However, even with the same fabricated condition, there is still device-to-device variation due to the difficult controllability of defects and the grain boundary of the advanced material-based sensing channel. Thus, during the sensor fabrication process, the defects and grain boundary should be considered in a microscopic regime, and a multi-sensor system study should be conducted at a macroscopic level to surpass these drawbacks. Moreover, well-controlled sensing materials also conserve low and stable background signals to achieve high and precise sensitivity. Furthermore, the integration of FET biosensors into an Internet of Things (IoT) device requires proper and secure information during signal modulation. Overall, solving these remaining issues makes the fabricated sensors more precise and reliable for commercialization. 

Like their other biosensor counterparts, FET biosensors are crucial in diagnosing and providing early warning for recognizing and treating AD. This study showed that an FET biosensor with a reasonable design based on advanced materials and cutting-edge technology proved capable of detecting AD biomarkers at the pico- or femtomolar LOD. However, certain necessary improvements to meet these requirements should be considered to obtain high selectivity and sensitivity. (1) A screening procedure for electrode fabrication should be considered to design an FET biosensor for AD biomarker detection. FET electrodes directly affect the performance of the device based on their characteristics, such as conductivity and contact point. Thus, the proper selection of materials and technology can enhance the sensitivity of the device. (2) To detect a specific AD biomarker, the selection of the bioreceptor is crucial. Each receptor is a specific analyte. Thus, a suitable selection of bioreceptors can be effective for target molecule recognition. (3) A combination of multiple approaches is necessary to achieve optimum performance. Based on sensor-immobilized biological probe characteristics, the appropriate utilization of multiple approaches results in a higher detection signal, thus improving device sensitivity. (4) The prediction of new circumstances by application of machine learning and deep learning should be conducted. Via technological development, the application of machine learning in biomedicine is of interest through collecting big data, training a model, and inferring a new circumstance by the manipulation of artificial intelligence (AI) power [120]. 

## Figures and Tables

**Table 1:** Summary of the FET biosensors for AD biomarker detection.

No.	Electrode Materials		Biofluids	Analyte	Linear Range	LOD	Ref.
	Source, Drain, Gate	Channel Layer					
1	Commercialized n-type FET chip	SiO_2_/APTES/GA/CR	HSA	Aβ_1-42_	10 pM–100 µM	NA *	[28]
2		SiO_2_/APTES/GA/Aβ_1-42_ Ab					
3	Ti/Au, Ti/Au, Ag/AgCl	Au/thiolate PEG (12 kDa)-COOH-EG_8_-thiol/EDC-NHS/Tau Ab/BSA	CSF	Tau	1 pM–10 nM	<1 pM	[34]
	Ti/Au, Ti/Au, Ag/AgCl	Au/thiolate PEG (20 kDa)-COOH-EG_8_-thiol/EDC-NHS/Tau Ab/BSA				<10 pM	
4	Ti/Pt, Ti/Pt, Ag/AgCl	G/PSE/Tau Ab/BSA	PBS, HS, and HP	Tau	10 fg/mL–1 ng/mL	NA	[29]
5	Ti/Au, Ti/Au, Ag/AgCl	Si/SiO_2_/rGO/PBASE/Tau Ab	PBS	Aβ_1-42_	1 pg/mL–100 ng/mL	NA	[33]
				t-Tau	100 fg/mL–1 ng/mL	NA	
			CSF, HP	Aβ_1-42_	100 fg/mL–100 ng/mL	222 fM	
				t-Tau	100 fg/mL–100 ng/mL	21.8 fM	
6	Au/Cr, Au/Cr, Organic semiconductor	Kapton/Au/PFBT/α-synuclein Ab	PBS	α-synuclein	0.25 pM to 25 nM	0.25 pM	[9]
7	Ti/Au, Ti/Au, Ag/AgCl	rGO/PBASE/AchE	PBS	Acetylcholine	1 µM to 10 mM	13.9 mV/dec	[8]
8	Cr/Au, Cr/Au, Cr/Au	Si/Al_2_O_3_/BG/p-tau Ab	PBS	p-tau_217_	10 fg/mL to 100 pg/mL	18.6 mV/dec	[66]
9			HSA			16.7 mV/dec	
10	NA	SiO_2_/PDOT: PSS/CR	PBS	Aβ_1-42_	2.21 pM–221 nM	216 μA/dec	[89]
11	Pb, Pb, Ag/AgCl	SiO_2_ NW/GPTES/ssDNA aptamer	PBS	Aβ_1-40_	0.1 pg/mL–10 µg/mL	20 fM	[92]
12	Au, Au, Ag wire	rGO/Au/NHS-EDC/Aβ_1-42_ Ab	PBS	Aβ_1-42_NDE–Aβ_1-42_	1.48–148 pg/mL	447 ag/mL	[94]
13	Ti/Pd/Au, Ti/Pd/Au, Y_2_O_3_	CNT/Au/DNA aptamer	Serum	Aβ_1-42_	1 fM to 10 pM	45 aM	[40]
14				Aβ_1-40_		55 aM	
15	Au/Al_2_O_3_, Au/Al_2_O_3_, top gate	Si/SiO_2_/APTES/rGO/RNA aptamer/BSA	PBS	Aβ_1-42_	1 ng/mL–1 pg/mL	NA	[93]
16	Cr/Au, Cr/Au,	CNT/sulfo-NHE/Ab/BSA	PBS	Aβ_1-42_	10^0^ to 10^6^ fM	2.13 fM	[90]
				Aβ_1-40_		2.20 fM	
				t-tau		2.45 fM	
				p-tau_181_		2.72 fM	

* NA: not available.
